# Abbreviated version of Penn State Worry Questionnaire for Chinese adolescents: Age, gender and longitudinal invariance

**DOI:** 10.3389/fpsyt.2023.1086592

**Published:** 2023-03-22

**Authors:** Shan-Shan Xie, Hui-Wen Xiao, Rong-Mao Lin

**Affiliations:** ^1^Department of Humanities and Social Sciences, Minjiang Teachers College, Fuzhou, Fujian, China; ^2^School of Psychology, Fujian Normal University, Fuzhou, Fujian, China; ^3^Department of Psychology, Renmin University of China, Beijing, China

**Keywords:** Penn State Worry Questionnaire (PSWQ), worry, measurement invariance, longitudinal measurement, adolescents

## Abstract

The abbreviated version of Penn State Worry Questionnaire (PSWQ-A) has been widely used to assess worry. However, its measurement invariance has been not yet warranted. With a cross-sectional and a longitudinal sample of Chinese adolescents (N1 = 1,329, N2 = 408), this study examined age, gender, and longitudinal invariance of PSWQ-A. Results supported strict invariance, including configural, metric, scalar, and error level, across gender and age in the cross-sectional sample; strict longitudinal measurement invariance was also supported in the longitudinal sample. This study suggests the application of the PSWQ-A in measuring adolescent worry and a basis for comparisons of different populations and occasions for worry.

## 1. Introduction

Excessive and uncontrollable worry, which refers to an anxious apprehension about future negative events, is common in and harmful to adolescents. Previous studies on adolescent worry have shown that more than 25% of adolescents report excessive and uncontrollable worry (1, 2). Worried adolescents not only frequently manifest inferior social and academic functioning, but also are at high risk for many mental disorders such as anxiety, depression, insomnia, etc. (3–5).

Considering the severity of adolescent worry, how to assess this issue has become an important topic in clinical and health research. The Penn State Worry Questionnaire (PSWQ) and the PSWQ-A (its abbreviated version) are considered the gold standard for assessing chronic worry, for they all enjoy excellent reliability and validity (6, 7). Similarly, the Penn State Worry Questionnaire for Children (PSWQ-C) is also a good instrument to assess trait worry in children and adolescents (8). However, compared to the full version of the PSWQ and PSWQ-C, the PSWQ-A not only includes fewer items (8 items *VS* 16 items/14 items) but also removes the impact of the method effect related to item wording (PSWQ has five negative worded items, and PSWQ-C has three negative worded items), and thus it is a superior option for researchers and clinical practitioners.

PSWQ-A has been examined in a variety of samples and was evidenced to have good psychometric properties in western countries. For example, Hopko et al. (6) proved that the PSWQ-A yielded high correlations with the PSWQ (*r* = 0.92), had good internal consistency (Cronbach’s alpha = 0.87), and demonstrated acceptable convergent validity with other anxiety measures (*r_s_* = 0.33–0.49) in a sample of senior citizens. Meanwhile, an increasing body of research supports the PSWQ-A, arguing that it also enjoys sound psychometric properties in adult and clinical samples. DeLapp et al. (9) demonstrated that in university students, the PSWQ-A enjoyed good internal consistency (Cronbach’s alpha = 0.92) and convergent validity with other measures of anxiety (*r_s_* = 0.47–0.52). Kertz et al. (10) found that in a clinical population, the total score of the PSWQ-A manifested good internal consistency (McDonald’s omega = 0.95) and convergent validity with measures of anxiety (*r_s_* = 0.39–0.68). However, PSWQ-A has been rarely used to assess adolescent worry, and whether the assessments of the PSWQ-A are made in the same way on multiple occasions in adolescent samples has been not yet sufficiently supported, according to reviewing the literature (9, 11). What’s more, previous studies on worry in children and adolescents were based on non-Chinese samples. Therefore, the main purpose of this study was to examine the age, gender, and longitudinal invariance of PSWQ-A for Chinese adolescents.

Measurement invariance (MI) refers to the relationships among latent variables and manifested indicators being invariant across occasions (12, 13), which means the person who has the same standing on the construct can receive the same observed score on the test. If the MI holds, a difference in the total scores for a given sample represents discrepancies in the construct of interest. If not, the observed changes may reflect differences in what is being measured rather than the latent construct (13). Although differences between subsamples of adolescent worry (e.g., gender, age) have been increasingly considered, the MI of the PSWQ-A for adolescents has yet to be tested.

### 1.1. Differences in worry across age

Cognitive maturation plays an important role in the development of worry (14, 15). An increasing body of evidence has shown that adolescents become more worried as they grow older. For example, Lin et al. (16) found that older adolescents (aged 16–18 years) reported higher levels of worry than did early adolescents (aged 13–15 years). Barahmand (17) also supported the conclusion that increasing age was linked to increased adolescent worry. However, do such differences stem from real differences between categories, or measurement bias? Reviewing previous literature, no research has explored the MI of the PSWQ-A across age groups or whether the PSWQ-A assesses the same constructs across all stages of puberty.

### 1.2. Differences in worry across gender

Numerous studies have indicated that adolescent worry differs significantly by gender. For example, Robichaud et al. (18) demonstrated that girls reported more worry than boys, specifically concerning a lack of confidence. This result was repeated in Chinese adolescents (16). However, Brown et al. (19) found that during puberty, boys tended to report more worry about the future than girls. Whether gender-based differences in adolescent worry result from the latent construct of worry or the measurement items remains uncertain.

### 1.3. Longitudinal MI

Longitudinal MI explores whether the same latent constructs are assessed over time within the same group, to ensure that changes in test scores over time can be attributed to actual changes in the construct under investigation (20, 21). Violation of longitudinal MI hampers the validity of score comparisons, especially in interventional studies. As MI is usually neglected when comparing different levels of worry in cross-sectional age groups, previous studies have focused little attention on longitudinal MI when comparing changes over time. For example, with a 10-wave longitudinal study of 338 adolescents, Dugas et al. (22) found that adolescent worry changed according to a curvilinear pattern. Anniko et al. (23) also concluded that worry significantly increased from early to middle adolescence. To some extent, these longitudinal differences reflect the development trend of adolescent worry but are susceptible to the interpretation of items representing that worry.

### 1.4. The current study

In summary, the PSWQ-A is a brief, convenient, and efficient measurement of adolescent worry. However, its MI across several occasions has been not yet supported in the adolescent sample. What’s more, the psychometric properties of PSWQ-A are rarely evidenced in Chinses samples. With a large cross-sectional sample of 1,329 Chinese adolescents and a longitudinal sample of 408 Chinese adolescents, therefore, this study examined the age, gender, and longitudinal MI for the PSWQ-A. Moreover, the incremental validity of the Ch-PSWQ-A was also tested using three criterion variables: stress, anxiety, and depression.

## 2. Method

### 2.1. Participants

Through cluster sampling, a sample of 1,329 adolescents was recruited from four middle schools in Fujian province, China. All participants completed questionnaires under the supervision of a head teacher and a postgraduate majoring in psychology. Approval was obtained from the participants’ head teacher and parents. After collecting questionnaires, participants responding with a large number of blanks or regular responses were excluded. Finally, an available sample of 1,229 adolescents was retained (the rate of availability was 92.5%). Participants ranged in age from 12 to 17 years (*M* = 14.3, SD = 1.5). According to previous guidelines (24), the age groups were divided into early (11–13 years old), moderate (14–15 years old), and late (16–17 years old) adolescence. Of the available sample, 599 were boys (48.7%) and 630 were girls (51.3%). The number of students in early, moderate, and late adolescence was 478, 395, and 349, respectively (seven participants did not report their age). This study was approved by the Academic Committee of **** University (masked for review).

A relatively moderately sized sample of 408 adolescents was separately recruited to test the longitudinal MI of the PSWQ-A. The participants completed the PSWQ-A twice, at the beginning and end of the first semester of the 2019–2020 academic year (Sept. 2019 and Jan. 2020). All participants were required a unique student number to match the two measurements. After collecting questionnaires, participants responding with a large number of blanks or regular were excluded. Finally, an available sample of 387 adolescents was retained (21 participants were excluded; the rate of exclusion was 5.1%, of which 12 were boys, seven were girls, and two did not report gender). Participants ranged in age from 11 to 16 years (*M* = 13.7, SD = 1.6). Of the available sample, 192 were boys (49.6%) and 195 were girls (50.4%).

### 2.2. Measures

#### 2.2.1. The abbreviated version of PSWQ-A

The PSWQ-A is an 8-item version of PSWQ that assesses a person’s tendency to worry independent of the topic of worry (6). Participants respond to each item by rating the frequency and severity of worry on a 5-point Likert scale ranging from 1 (not at all typical of me) to 5 (very typical of me). The total score of the PSWQ-A ranges from 8 to 40, with higher scores indicating greater levels of worry. The reliability and validity of the PSWQ-A were sound in adult and clinical samples (9, 25). The Chinese version of the PSWQ-A was simplified from the full Chinese version of PSWQ, which showed good reliability and validity (26). This study further tested its psychometric properties and MI in Chinese adolescents.

#### 2.2.2. Depression-anxiety-stress scale, DASS-21

The DASS-21 is a 21-item self-report measure, including stress, anxiety, and depression subscales (27). Participants respond to each item by rating the frequency and severity of symptoms experienced during the previous week on a 4-point Likert scale ranging from 0 (does not apply to me at all) to 3 (applies to me very much or most of the time). The total score of the DASS-21 ranges from 0 to 63, and the scores for stress, anxiety, and depression range from 0 to 21. Higher scores indicate higher levels of negative emotionality. The Chinese version of the DASS-21 and its subscales showed good internal consistency (Cronbach’s alpha = 0.89, as do the subscales of stress, anxiety, and depression, 0.76, 0.79, and 0.77, respectively), construct validity, and convergent and discriminate validity (28). In the present study, Cronbach’s alpha coefficient of the DASS-21 was 0.94, and the value for the stress, depression, and anxiety subscales were 0.85, 0.85, and 0.88, respectively.

### 2.3. Data analysis strategy

All data were analyzed using SPSS 25.0 and Mplus 8.0 for Windows. First, a CFA of a unidimensional factor model was performed to examine the construct validity. Second, following the previous relevant studies (29), the gender and age MI were tested using the large cross-sectional sample, and the longitudinal MI was evaluated *via* the longitudinal sample. The unidimensional factor model was first separately tested for the subsamples, and then four restrictive models were used to test for (a) configural invariance (equal form), (b) metric invariance (equal factor loadings), (c) scalar invariance (equal indicator thresholds), and (d) error invariance (equal indicator residual errors) (12, 13). Finally, a structural regression model was employed to examine whether the PSWQ–A was positively correlated with stress, anxiety, and depression, further evidencing the incremental validity of the instrument.

The overall fit of the CFA and MI models was evaluated using a series of goodness-of-fit statistics and applicability-of-model parameters. The Chi-squared statistic (*χ^2^*), normed Chi-squared statistic (*χ^2^/df*), and multiple complementary fit indices were considered. When evaluating model fit, *χ^2^* is non-significant, and the value of *χ^2^/df* is less than 5, which usually suggests an acceptable model (30). However, *χ^2^* and *χ^2^/df* are significantly influenced by sample sizes (31). Multiple complementary fit indices, including the Tucker-Lewis Index (TLI), Comparative Fit Index (CFI), Root Mean Square Error of Approximation (RMSEA), and Standardized Root Mean Square Residual (SRMR) were also used to evaluate the model fit (32). The CFI and TLI measure how much better the selected model is than the baseline model, and values above 0.90 are considered to suggest an acceptable model fit (31). Similarly, the value of RMSEA and SRMR should be less than 0.08 (31). In the present research, differences in overall Chi-squared value and related degrees of freedom (*Δχ^2^, Δχ^2^/Δdf*) were considered when evaluating the MI across gender, age, and time. In addition, a change in CFI (ΔCFI) is recommended. A ΔCFI value less than or equal to 0.01 is evidence of parameter invariance between groups (33, 34).

## 3. Results

### 3.1. Confirmatory factor analysis, CFA

A CFA was conducted to examine the unidimensional factor model of the PSWQ-A. The fitting indexes were as follows: *χ^2^* = 290.686, *df* = 20, *χ^2^/df* = 14.534, *RMSEA* = 0.109, *CFI* = 0.935, *TLI* = 0.909, *SRMR* = 0.038. According to the value of the modification index, two residual error correlations (Items 6 with 7 and 12 with 13) were specified. Except for the value of *χ^2^* still being significant and *χ^2^*/*df* greater than 5 (*χ^2^* (18) = 122.409, *χ^2^*/*df* = 6.801), the other fit indices were at acceptable levels (*CFI* = 0.975, *TLI* = 0.961, *RMSEA* = 0.071, and *SRMR* = 0.026). Thus, the modified unidimensional factor model of the PSWQ-A was acceptable and the construct validity was supported.

### 3.2. Internal reliability

Cronbach’s alphas were computed in the present study to assess the internal consistency of the PSWQ-A. The results showed that Cronbach’s alpha was 0.89 in the cross-sectional sample. In addition, Cronbach’s alphas were 0.84 and 0.88 at time points 1 and 2 in longitudinal samples. It demonstrated that PSWQ-A had satisfactory internal consistency.

### 3.3. Gender MI

As shown in Table 1, the PSWQ-A model showed a good fit between both boys and girls (Models 1 and 2), which was a prerequisite to testing the measurement invariance across gender. The model testing for configural invariance (Model 3) achieved a good fit, indicating that both girls and boys possessed the same structures for the PSWQ-A. Following this, all factor loadings were constrained to be equal across gender (Model 4). Comparing Models 3 and 4, the Chi-squared difference was not significant (*p* = 0.519) and the ΔCFI test was below the 0.01 cutoff (0.001), supporting metric invariance across girls and boys. Model 5 added constraints to the intercepts, resulting in a significant *Δχ^2^* of 27.578 for 7 *df* (*p* < 0.001). However, the value of *Δχ^2^*/*Δdf* was less than 5 (3.940), and the ∆CFI test was below the 0.01 cutoff (0.006). This supported scalar invariance across girls and boys. Model 6 constrained the error variances to be equivalent across gender, resulting in a significant *Δχ^2^* of 20.952 for 8 *df* (*p* < 0.001). However, the value of *Δχ^2^/Δdf* (2.619) was less than 5 and a low ΔCFI (0.004 < 0.01) supported error invariance across girls and boys. Thus, a strict MI across gender was supported.

**Table 1:** Measurement invariance of the PSWQ-A across gender, age and times.

Model	*χ^2^*	*df*	*χ^2^*/*df*	*CFI*	*TLI*	*RMSEA*	*SRMR*	Model comparison	*Δχ^2^(Δdf)*	*Δχ^2^/Δdf*	*Δ CFI*
Measurement invariance across gender (*n* = 1229)											
Model 1: Model 1for boys (*n* = 599)	52.736	18	2.930	0.977	0.964	0.059	0.030	–	–	–	–
Model 2: Model 2 for girls (*n* = 630)	61.611	18	3.423	0.975	0.962	0.065	0.029	–	–	–	–
Model 3: Configural invariance	114.291	36	3.175	0.976	0.963	0.062	0.029	–	–	–	–
Model 4: Metric invariance	125.639	43	2.922	0.975	0.967	0.058	0.033	3*VS*4	6.183 (7)	0.883	−0.001
Model 5: Scalar invariance	151.736	50	3.035	0.969	0.965	0.060	0.039	4*VS*5	27.578 (7)	3.940	−0.006
Model 6: Error invariance	172.851	58	2.980	0.965	0.966	0.059	0.041	5*VS*6	20.952 (8)	2.619	−0.004
Measurement invariance across age (*n* = 1222^&^)											
Model 7: Model for age 12–13 (*n* = 478)	49.414	18	2.745	0.976	0.963	0.060	0.028	–	–	–	–
Model 8: Model for age 14–15 (*n* = 395)	27.463	18	1.526	0.992	0.988	0.036	0.024	–	–	–	–
Model 9: Model for age 16–17 (*n* = 349)	38.104	18	2.117	0.981	0.970	0.057	0.032	–	–	–	–
Model 10: Configural invariance	114.734	54	2.125	0.983	0.973	0.053	0.028	–	–	–	–
Model 11: Metric invariance	134.403	68	1.977	0.981	0.977	0.049	0.038	10*VS*11	14.593 (14)	1.042	−0.002
Model 12: Scalar invariance	162.355	82	1.980	0.977	0.977	0.049	0.041	11*VS*12	28.025 (14)	2.001	−0.004
Model 13: Error invariance	213.488	98	2.178	0.967	0.972	0.054	0.050	12*VS*13	51.801 (16)	3.238	−0.010
Longitudinal measurement invariance (*n* = 387)											
Model 14: Model for time 1	47.281	18	2.627	0.964	0.944	0.067	0.039	–	–	–	–
Model 15: Model for time 2	58.506	18	3.250	0.962	0.940	0.077	0.036	–	–	–	–
Model 16: Configural invariance	189.783	91	2.086	0.953	0.938	0.055	0.044	–	–	–	–
Model 17: Metric invariance	210.742	98	2.150	0.946	0.934	0.056	0.055	16*VS*17	23.749 (7)	3.393	−0.007
Model 18: Scalar invariance	240.426	105	2.290	0.936	0.926	0.060	0.058	17*VS*18	31.963 (7)	4.566	−0.010
Model 19: Error invariance	268.153	113	2.373	0.926	0.922	0.062	0.058	18*VS*19	30.380 (8)	3.797	−0.010

RMSEA, root mean squared error of approximation; SRMR, standardized root mean square residual; CFI, Comparative fit index. TLI, Tucker-Lewis index.

& seven participants did not report their age and thus did not enter age measurement.

### 3.4. Age MI

As shown in Table 1, the fit indices of the PSWQ-A model for the three age groups (Models 7, 8, and 9) were all good, satisfying the premise of MI. The model testing for configural invariance (Model 10) met the specified guidelines, indicating that the three age groups possessed the same structures for the PSWQ. Model 11 added constraints to the factor loading to be equivalent across ages. The results showed that the value of *Δχ^2^* was non-significant (14.593, *p* = 0.407), *Δχ^2^/Δdf* (1.042) was less than 5 and Δ CFI was less than 0.01 (0.002), supporting the metric invariance. Model 12 constrained the intercepts to be equivalent across age, resulting in a significant *Δχ^2^* of 28.025 for 14 *df* (*p* < 0.001). However, lower *Δχ^2^/Δdf* and ΔCFI (2.001 < 5 and 0.004 < 0.01, respectively) supported scalar invariance across age. Model 13 made the constraint residual error equivalent. The results showed a significant *Δχ^2^* (*p* < 0.001), but *Δχ^2^/Δdf* (3.238 < 5) was lower and ΔCFI was equal to 0.01, supporting error invariance across age. Thus, the MI of the PSWQ-A across ages also achieved strict invariance.

### 3.5. Longitudinal MI

The PSWQ-A model showed good model fits for Time 1 and 2 (Models 14 and 15), which was a prerequisite for testing MI across time. The model testing for configural invariance (Model 16) achieved a good model fit, indicating that Time 1 and 2 possessed the same structures for the PSWQ-A. Following this, all factor loadings were constrained to be equal across time (Model 17). Comparing Models 16 and 17 showed that the value of *χ^2^* significantly increased (*p* = 0.001). However, *Δχ^2^*/*Δdf* was less than 5 (3.393) and the CFI decreased to less than 0.01 (0.007), supporting metric invariance across Time 1 and 2. Model 18 added constraints to the intercepts, resulting in a significant *Δχ^2^* of 31.963 for 7 *df* (*p* < 0.001). However, the *Δχ^2^*/*Δdf* was less than 5 (4.566) and the CFI decreased to be equal to 0.01, supporting scalar invariance across Time 1 and 2. Model 19 constrained the error variances to be equivalent across time, resulting in a significant *Δχ^2^* of 30.380 for 8 *df* (*p* < 0.001). However, the value of *Δχ^2^/Δdf* (3.797) was less than 5 and ΔCFI was equal to 0.01, supporting error invariance across Time 1 and 2. Thus, a strict MI across time was supported, as well.

### 3.6. Structural regression model

**Figure 1:**
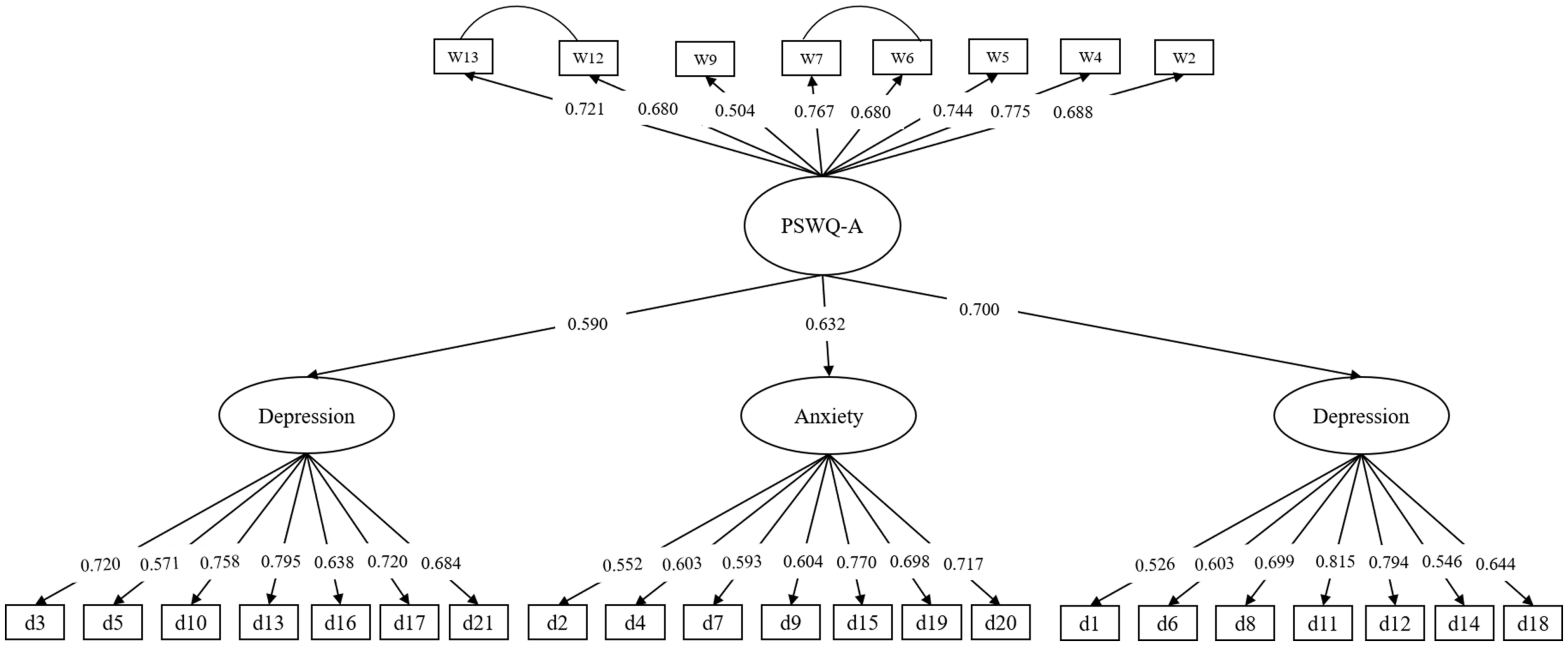
Standardized path values for the structural regression model.

The structural regression model provided an acceptable fit to the data (*χ2* = 1,813.292, *df* = 369, *χ^2^/df* = 4.914, *RMSEA* = 0.059, *CFI* = 0.915, *TLI* = 0.907, and *SRMR* = 0.046). The loadings of all variables for structural regression model were significant (in Figure 1). Worry was significantly associated with the subscales of depression, anxiety, and stress. Percentages of variance explained by the PSWQ-A in depression, anxiety, and stress were 35, 40, and 49%, respectively.

## 4. Discussion

To our knowledge, this study was the first to fully examine the MI of the PSWQ-A across gender, age, and time. After a series of investigations, this study validated the unidimensional solution of the PSWQ-A in Chinese adolescent samples. Furthermore, the results from multiple-group CFA showed that the PSWQ-A demonstrated not only strict temporal MI across gender and age groups, but also strict longitudinal MI across two time periods (the beginning and end of a semester). Moreover, good incremental validity was also demonstrated, with positive correlations with indexes of depression, anxiety, and stress. This study supports a strict MI of the PSWQ-A in Chinese adolescents, warranting the application of the PSWQ-A in measuring adolescent worry.

Firstly, the unitary construct of the PSWQ-A is validated in Chinese adolescents. With the removal of the five negatively worded and three positively worded items, the PSWQ-A not only eliminates the need to account for the method effect (25), but also effectively accelerates the application of the measurement of worry. And its construct validity has been also sufficiently supported in adult and clinical samples (6, 35). For the Chinese adolescents, a modified unitary construct that specified two pairs of items’ residual error corrected (items 6 with item 7, item 12 with item 13) fit the data very well. The less-than-perfect construct model in this study may result from the large sample, for the chi-square test and the RMSEA are significantly influenced by the sample sizes (31, 36), and it also may result from a serial position effect that the participants answered the PSWQ-A item from the first to the last (37). Two pairs of items’ residual error significant correlations warm us that more shorten version of the PSWQ may be feasible, for the corrected items can be only retained as one in the measure (38).

Furthermore, the findings of the current study supported configural, metric, scalar, and strict invariance for the PSWQ-A across gender, age, and time, which provided evidence that the measurement invariance requirement for valid group comparisons has been satisfied. It means worry, which was assessed by PSWQ-A, has the same meaning for boys and girls and early, moderate, and late adolescents, as well as longitudinally across two time periods. According to previous literature, when the MI is established, it is entitled to state that differences observed are actual differences between populations and not artifacts of measurement (12, 13). As a consequence, different worry levels by gender and development period as measured using PSWQ-A can be considered to reflect actual group differences, and not biased assessments.

Moreover, the PSWQ-A also demonstrated good incremental validity in Chinese adolescents. The PSWQ-A positively correlated with depression, anxiety, and stress, and explained 35, 40, and 49% of the variance in depression, anxiety, and stress, respectively. This result also supports the core role of worry, leading to health problems related to stress and negative emotions (39–41).

In total, this study supports the application of the PSWQ-A in measuring adolescent worry and it is served as the basis for comparisons of different populations and occasions for worry. Several limitations should also be considered. First, this research only sampled normal Chinese adolescents and not those receiving clinical treatment (especially for GAD). Thus, verification of the sound validity and reliability of the PSWQ-A is restricted to when the measure is being used for normal adolescents. Future work should consider extending the results to adolescents being clinically treated for GAD and testing the MI between the normal and clinical groups. Similarly, since PSWQ-A is more widely used abroad and rarely in China, it is possible to compare the differences in worry levels at home and abroad in the future. Second, all data in this study were collected by self-reported, and thus some bias in the results could be found. Future research should employ multiple assessors (e.g., teachers, fathers, and/or mothers) to avoid common measure bias. Moreover, limited criteria indexes that only included depression, anxiety, and stress were used to test the incremental validity. Future work should adopt more variables and measures to extend the incremental validity of the PSWQ-A.

## Data availability statement

The original contributions presented in the study are included in the article/supplementary material, further inquiries can be directed to the corresponding author.

## Ethics statement

The studies involving human participants were reviewed and approved by The School of Psychology, Fujian Normal University. Written informed consent to participate in this study was provided by the participants' legal guardian/next of kin.

## Author contributions

All the authors participated in the conception and design of the work. Specifically, R-ML conceived the original idea for the study. And the study paper was written by S-SX and H-WX, therefore both of them were the first co-authors. R-ML supervised this study. All authors contributed to the article and approved the submitted version.

## Conflict of interest

The authors declare that the research was conducted in the absence of any commercial or financial relationships that could be construed as a potential conflict of interest.

## Publisher’s note

All claims expressed in this article are solely those of the authors and do not necessarily represent those of their affiliated organizations, or those of the publisher, the editors and the reviewers. Any product that may be evaluated in this article, or claim that may be made by its manufacturer, is not guaranteed or endorsed by the publisher.
